# Reactivities of a Prostanoid EP2 Agonist, Omidenepag, Are Useful for Distinguishing between 3D Spheroids of Human Orbital Fibroblasts without or with Graves’ Orbitopathy

**DOI:** 10.3390/cells10113196

**Published:** 2021-11-16

**Authors:** Yosuke Ida, Hanae Ichioka, Masato Furuhashi, Fumihito Hikage, Megumi Watanabe, Araya Umetsu, Hiroshi Ohguro

**Affiliations:** 1 Departments of Ophthalmology, School of Medicine, Sapporo Medical University, Sapporo 060-8556, Japan; ichioka29@sapmed.ac.jp (H.I.); fuhika@gmail.com (F.H.); watanabe@sapmed.ac.jp (M.W.); araya.alaya.favreweissth@gmail.com (A.U.); ooguro@sapmed.ac.jp (H.O.); 2 Department of Cardiovascular, Renal and Metabolic Medicine, School of Medicine, Sapporo Medical University, Sapporo 060-8556, Japan; furuhasi@sapmed.ac.jp

**Keywords:** prostanoid EP2 agonist, ROCK inhibitor, three-dimension (3D) cell culture, Graves’ orbitopathy, IGF-1, orbital fibroblast

## Abstract

Background. To obtain new insights into the activation of the thyroid-stimulating hormone (TSH) and insulin-like growth factor 1 (IGF-1) receptors in human orbital fibroblasts (n-HOFs), the effects of the prostanoid EP2 agonist, omidenepag (OMD), and a rho-associated coiled-coil-containing protein kinase (ROCK) inhibitor, ripasudil (Rip) were evaluated using three-dimension (3D) n-HOFs spheroids in the absence and presence of the recombinant human TSH receptor antibodies, M22 and IGF-1. Methods. The effects of 100 nM OMD or 10 μM Rip on the physical properties, size, stiffness, and mRNA expression of several extracellular matrix (ECM) molecules, their regulator, inflammatory cytokines, and endoplasmic reticulum (ER) stress-related factors were examined and compared among 3D spheroids of n-HOFs, M22-/IGF-1-activated n-HOFs and GO-related human orbital fibroblasts (GHOFs). Results. The physical properties and mRNA expressions of several genes of the 3D n-HOFs spheroids were significantly and diversely modulated by the presence of OMD or Rip. The OMD-induced effects on M22-/IGF-1-activated n-HOFs were similar to the effects caused by GHOHs, but quite different from those of n-HOFs. Conclusions. The findings presented herein indicate that the changes induced by OMD may be useful in distinguishing between n-HOFs and GHOFs.

## 1. Introduction

Graves’ orbitopathy (GO), an autoimmune disease that affects orbital and periorbital tissues, demonstrates several characteristic clinical manifestations, including upper eyelid retraction, edema, and erythema of the periorbital tissues and conjunctivae, as well as exophthalmos [1,2]. Regarding the molecular pathology of GO, autoimmune responses to the thyroid-stimulating hormone receptor (TSHR) induced the inflammation of orbital fatty tissues resulting in the unfavorable remodeling of the extracellular matrix (ECM) [3,4]. 

Among the currently available anti-glaucoma medications, prostaglandin analogues (PGs) are used as a first-line medication based upon their strong efficacy in lowering IOP, as well as having less systemic side effects. However, local side effects, which are referred to as “the deepening of the upper eyelid sulcus (DUES)”, are now recognized as cosmetically non-negligible [5]. The induction of orbital fat atrophy, induced by PGs, is proposed as a possible etiology, based on several clinical studies as well as in vitro studies [6]. Alternatively, we speculate that such effects induced by these PGs that cause orbital fat atrophy may be beneficial for increased GO-related orbital fat. However, a previous randomized, controlled, double-masked crossover trial indicated that a bimatoprost (BIM) treatment over a three-month period failed to result in an improvement in proptosis in GO [7]. Therefore, these collective results indicate that the PG-induced periocular effects on orbital fatty tissues are different between GO and non-GO, simultaneously suggesting that other anti-glaucoma drugs could be considered as candidates for this condition. 

The possibility that anti-glaucoma medications might induce periocular side effects has attracted considerable interest. This is especially the case for new types of the anti-glaucoma medications, such as the selective, non-prostaglandin, prostanoid EP2 agonist prodrug, omidenepag isopropyl (OMDI); and the Rho-associated coiled-coil-containing protein kinase (ROCK) inhibitor, ripasudil hydrochloride hydrate (Rip) [8,9], since these drugs have distinctly different pharmacological properties compared to PGs. To examine this issue in more depth, using 3D spheroid cultures [10], we compared the effects of several anti-glaucoma drugs including a prostaglandin F2α analogue (bimatoprost acid; BIM-A), an EP2 agonist (OMD) and a ROCK inhibitor (Rip) on GO-related human orbital fibroblasts (GHOFs) [11]. The findings indicate that these anti-glaucoma drugs modulate both the structures and physical properties of 3D GHOFs spheroids in different manners by modifying the gene expressions of ECM, ECM-regulatory factors and inflammatory cytokines. Among these drug-induced effects, OMD and Rip caused a significant increase in the sizes of the 3D GHOFs spheroids and the BIM-A-induced substantial downsizing effects [11]. Since it was revealed that the simultaneous activation of TSH and IGF-1 receptors were required for GO pathogenesis, the studies of these OMD- or Rip-induced effects on non-GO-related human orbital fibroblasts (n-HOFs) and TSH and IGF-1 receptor-stimulated n-HOFs were expected to shed more light on this issue.

Therefore, in the current study, to elucidate the effects of OMD or Rip on n-HOFs and TSH, as well as IGF-1 receptor-activated n-HOFs, n-HOFs were obtained from patients with orbital fat herniations without GO and cultured in the form of 3D drop cultures [10,12] in the presence or absence of a recombinant human TSH receptor antibody, M22 and IGF-1 [13,14,15]. The pharmacopathological effects on these 3D n-HOFs spheroids were then examined, and the effects of these preparations were compared with our previous study using GHOFs [11]. 

## 2. Materials and Methods

All procedures in the current study involving human participants were conducted at Sapporo Medical University Hospital, Japan, and approved by the institutional review board (approved number, 312-3190) according to the tenets of the Declaration of Helsinki and the national laws for the protection of personal data. Informed consent was obtained from all individual participants included in this study.

### 2.1. Isolation and 3D Cultures of Human Orbital Fibroblasts (HOFs) without Graves’ Orbitopathy (GO) (n-HOFs) 

Isolation of n-HOFs was performed by a previously described method using surgically obtained orbital fat explants from 4 non-GO patients with orbital fat herniation. In the absence and presence of 10 ng/mL M22 and 100 ng/mL IGF-1, their three-dimensional (3D) spheroid cultures were then processed during 6 days as described in a recent report [11,12,16]. These M22 and IGF-1 concentrations were based on data reported in previous studies [13,14,15].

For evaluation of the pharmacological efficacy of 100 mM omidenepag (OMD, a generous gift from Santen Pharmaceutical Co., Ltd., Osaka, Japan), a prostanoid EP2 agonist, or 10 µM ripasudil (Rip, a generous gift from the Kowa Company Ltd., Nagoya, Japan), a ROCK inhibitor, using the optimum concentrations confirmed in earlier studies [17,18], were added during Day 1 through Day 5. 

The 3D spheroid configuration was observed by phase contrast (PC, Nikon ECLIPSE TS2; Tokyo, Japan) as described previously [10]. For measurement of the size of each 3D spheroid, the largest cross-sectional area (CSA) of the PC image was measured and analyzed by the Image-J software version 1.51n (National Institutes of Health, Bethesda, MD, USA) [10].

### 2.2. Micro-Indentation Force Measurement

The micro-indentation force (μN) required to achieve a 50% deformation of the HOFs spheroids during 20 s was measured using a micro-squeezer (CellScale, Waterloo, ON, Canada) as described previously and Force/displacement (μN/μm) was calculated [10].

### 2.3. Quantitative PCR

Total RNA was extracted from 3D n-HOF spheroids under several conditions as above using a RNeasy mini kit (Qiagen, Valencia, CA, USA) and following reverse transcription by the SuperScript IV kit (Invitrogen, Carlsbad, CA, USA), were processed according to the manufacturer’s instructions. In addition to these n-HOF-related cDNAs, previously prepared cDNAs obtained from GHOF spheroids [11] were subjected to real-time PCR with the Universal Taqman Master mix using a StepOnePlus machine (Applied Biosystems/Thermo Fisher Scientific, Carlsbad, MA, USA). cDNA levels expressed as fold-change relative to the expression of a housekeeping 36B4 (*Rplp0*) gene were then calculated. The sequences of the primers and Taqman probes used are shown in Table 1.

### 2.4. Statistical Analysis

All statistical analyses were performed using the Graph Pad Prism 8 (GraphPad Software, San Diego, CA, USA). To analyze the difference between groups, a grouped analysis with a two-way analysis of variance (ANOVA) followed by a Tukey’s multiple comparison test was performed. Data are presented as the arithmetic mean ± the standard error of the mean (SEM).

## 3. Results

### 3.1. Effects of an EP2 Agonist, OMD and the ROCK Inhibitor, Rip on Physical Properties, Size and Stiffness of the 3D Spheroid Obtained from Non-GO-Related Human Orbital Fibroblast (n-HOFs) and GO-Related Human Orbital Fibroblast (GHOF)

**Figure 1:**
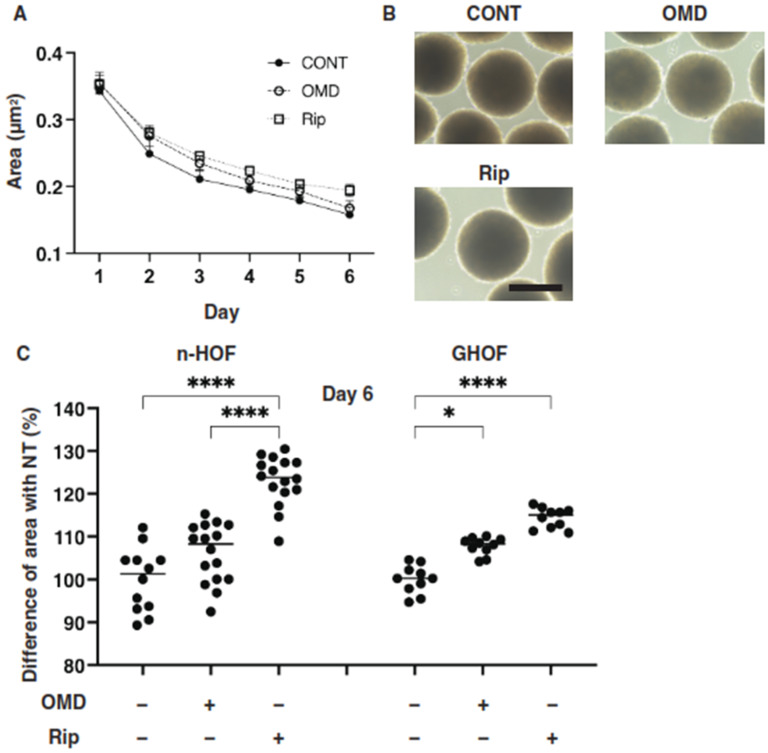
Effects of omidenepag (OMD) and ripasudil (Rip) on the mean sizes of n-HOFs or GHOF 3D spheroids. Panel (**A**) Changes in the mean area sizes (μm^2^) of the 3D spheroids derived from n-HOFs cells cultured without (CONT, closed circles) or with 100 nM omidenepag (OMD, closed squares) or 10 µM Ripasudil (Rip, closed triangles) were plotted during the 6-day culture period. Panel (**B**) Representative phase contrast images of the 3D n-HOFs spheroids under several conditions as above at Day 6 are shown (scale bar; 100 μm). Panel (**C**) Percentage difference in the size of the mean area of n-HOF or GHOF spheroids treated with omidenepag (OMD) or ripasudil (Rip) as above, as compared with their non-treated control (CONT), was plotted. In terms of the results for the GHOF spheroids, corresponding data reported in our previous study [11] were recalculated and replotted. All experiments were performed in triplicate using fresh preparations, each consisting of 16 spheroids. Data are presented as the arithmetic mean ± the standard error of the mean (SEM). * *p* < 0.05, **** *p* < 0.001 (ANOVA followed by a Tukey’s multiple comparison test).

**Figure 2:**
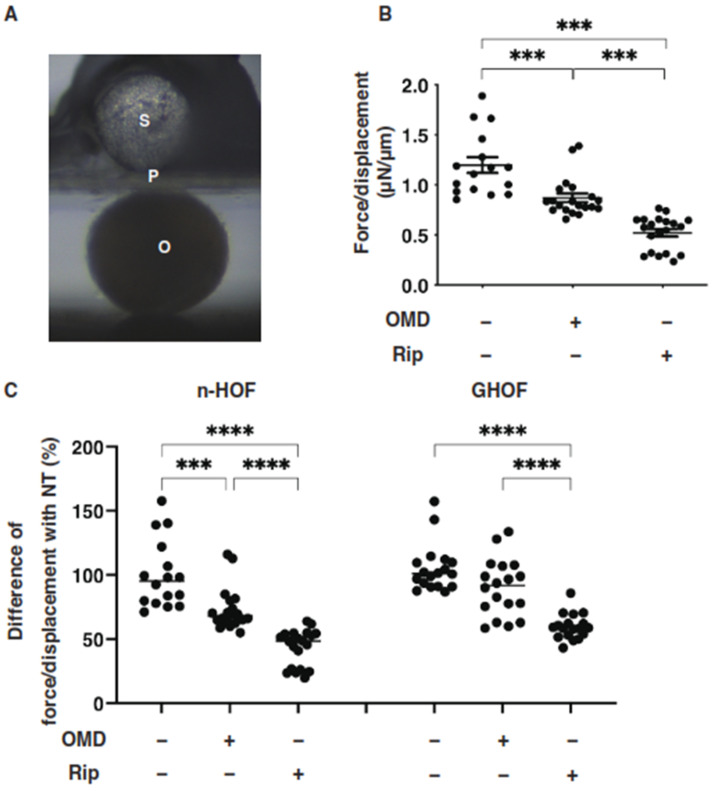
Effects of omidenepag (OMD) or ripasudil (Rip) on the physical stiffness of n-HOFs or GHOF 3D spheroids. At Day 6, the n-HOFs 3D spheroids without or with 100 nM omidenepag (OMD) or 10 µM Ripasudil (Rip) were subjected to a physical solidity analysis by a micro-squeezer (panel (**A**): S—pressure sensor, P—plate, O—3D HOFs spheroid). The force required to induce deformation until half diameter was reached (μN/μm force/displacement) was measured and plotted (panel (**B**)). Percentage difference in the force/displacement values of n-HOF or GHOF spheroids treated with omidenepag (OMD) or ripasudil (Rip) as above, as compared with their non-treated control (CONT), was plotted (panel (**C**), left). In terms of the results of the GHOF spheroids, the corresponding data reported in our precedent study [11] were recalculated and replotted (panel (**C**), right). All experiments were performed using freshly prepared 12–20 spheroids. *** *p* < 0.005, **** *p* < 0.001 (ANOVA followed by a Tukey’s multiple comparison test).

Several studies reported that anti-glaucoma medications for PGs induced significant effects on peri-ocular tissues such as orbital fat atrophy [19,20,21], a condition that is referred to as “DUES”. In our previous study, we reported that new types of anti-glaucoma drugs, including an EP2 agonist, OMD, and the ROCK inhibitor, Rip, induced different effects from those of the PGs in 3D spheroids obtained from GO-related fibroblasts (GHOF) [11]. To study this issue in more detail, the effects of these drugs on non-GO-related human orbital fibroblasts (n-HOF) were investigated using 3D spheroid cultures derived from the fibroblasts. Similar to the findings reported in our previous studies [12,16], uniform round-shaped 3D spheroids from 20,000 n-HOFs cells grew into smaller and matured forms during the 6-day culture (Figure 1A,B). In the presence of 100 nM OMD, the physical properties were different between the 3D n-HOF (size: no significant change, stiffness: marked decrease) and GHOF spheroids (size: substantial enlargement, stiffness: no significant change), although the Rip-induced effects on 3D n-HOFs spheroids were similar to those of the 3D GHOF spheroids (size: substantial enlargement, stiffness: significant decrease) [11] (Figure 1A,C and Figure 2). 

### 3.2. The Effects of an EP2 Agonist, OMD, and the ROCK Inhibitor, Rip, on mRNA Expression of ECM, ECM Regulatory Genes and Inflammatory Cytokine of the 3D Spheroid Obtained from Non-GO-Related Human Orbital Fibroblast (n-HOFs) and GO-Related Human Orbital Fibroblast (GHOF)

**Figure 3:**
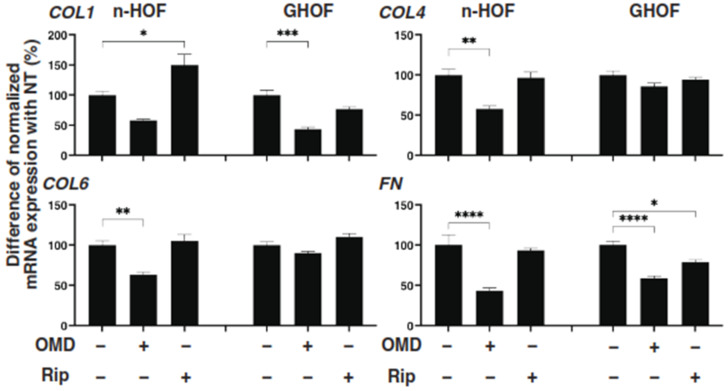
mRNA expression of ECMs in n-HOFs or GHOF 3D spheroids under several conditions. At Day 6, the n-HOFs 3D spheroids without or with 100 nM omidenepag (OMD) or 10 µM Ripasudil (Rip) were subjected to a qPCR analysis to estimate the mRNA expression of ECMs (*COL1*: collagen 1, *COL4*: collagen 4, *COL6*: collagen 6, *Fn*: fibronectin). Percentage difference in the mRNA expressions of these respective genes of n-HOF or GHOF spheroids treated with omidenepag (OMD) or ripasudil (Rip) as above, as compared with their non-treated control (CONT), were plotted. In terms of the results of the GHOF spheroids, corresponding data reported in our previous study [11] were recalculated and replotted. All experiments were performed in duplicate using fresh preparations, each of which consisted of 16 spheroids. Data are presented as the arithmetic mean ± standard error of the mean (SEM). * *p* < 0.05, ** *p* < 0.01, *** *p* < 0.005, **** *p* < 0.001 (ANOVA followed by a Tukey’s multiple comparison test).

**Figure 4:**
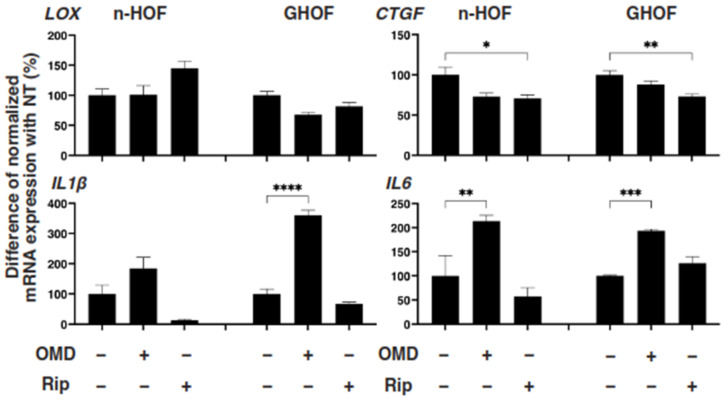
mRNA expression of ECM-regulatory genes and inflammatory cytokines in n-HOFs or GHOF 3D spheroids under several conditions. At Day 6, the n-HOFs 3D spheroids without or with 100 nM omidenepag (OMD) or 10 µM Ripasudil (Rip) were subjected to a qPCR analysis to estimate the mRNA expression of ECM-regulatory genes (*LOX*: lysil oxidase, *CTGF*: Connective Tissue Growth Factor, *EPAS1*: endothelial PAS domain-containing protein 1), and inflammatory cytokines (*IL1β*: interleukin-1β, *IL6*: interleukin-6). Percent difference in the mRNA expressions of these respective genes of n-HOF or GHOF spheroids treated with omidenepag (OMD) or ripasudil (Rip) as above, as compared with their non-treated control (CONT) were plotted. In terms of the results of the GHOF spheroids, corresponding data reported in our previous study [11] were recalculated and replotted. All experiments were performed in duplicate using fresh preparations, each of which consisted of 16 spheroids. Data are presented as the arithmetic mean ± standard error of the mean (SEM). * *p* < 0.05, ** *p* < 0.01, *** *p* < 0.005, **** *p* < 0.001 (ANOVA followed by a Tukey’s multiple comparison test).

To further study these OMD- or Rip-induced effects on the 3D n-HOFs and GHOFs spheroids, we examined these drug-induced effects on the mRNA expression of the major ECMs of the 3D n-HOFs spheroid. These included *collagen1* (*COL1*), *COL4*, *COL6*, and *fibronectin* (*FN*) (Figure 3); ECM-regulatory genes, including *lysil oxidase* (*LOX*), *Connective Tissue Growth Factor* (*CTGF*) and *endothelial PAS domain-containing protein1* (*EPAS1*); and inflammatory cytokines including *interleukin-1β* (*IL1β*) and *interleukin-6* (*IL6*) (Figure 4). In the n-HOFs, the mRNA expression of all four ECMs, except *COL1* (Figure 3) or *IL6* (Figure 4), were down-regulated or up-regulated, respectively, in the presence of OMD, while, in contrast, the significant down-regulation of *COL1* and *FN*, and up-regulation of *IL1β* and *IL6*, were observed in the GHOFs. In the presence of Rip, *COL1* and *CTGF* were significantly up-regulated and down-regulated, respectively, in the n-HOFs, and *FN* and *CTGF* were substantially down-regulated in the GHOFs (Figure 4). These collective findings suggest that both OMD and Rip significantly and differently alter the physical properties and the gene expressions of the ECMs, their regulatory factors and the inflammatory cytokines of the 3D n-HOFs spheroids. Interestingly, the OMD-induced effects on most of the gene expressions, including on *COL1*, *COL4*, *COL6*, *LOX*, *CTGF* and *IL1β*, were different compared to those on 3D GHOFs spheroids (summarized in Table 2). Therefore, considering these collective findings, we concluded that drug efficacy by OMD may be a useful indicator for distinguishing 3D n-HOFs spheroids from 3D GHOFs spheroids. 

### 3.3. Effects of TSH Receptor Stimulation by M22, and/or IGF-1 Receptor Stimulation of 3D n-HOFs Spheroid on the Physical Properties in the Presence of OMD

**Figure 5:**
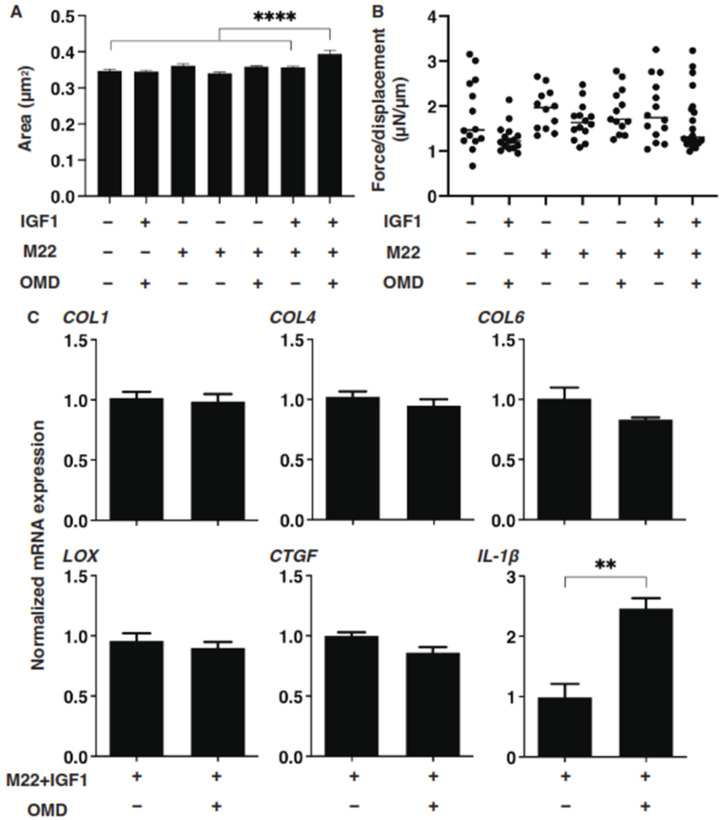
Effects of IGF-1, M22 and/or omidenepag (OMD) on the physical properties, size and stiffness of the n-HOFs 3D spheroids. At Day 6, n-HOFs 3D spheroids were treated with 100 ng/mL IGF-1,10 ng/mL M22 and/or 100 nM omidenepag (OMD), and their mean sizes and stiffness (μN/μm force/displacement) were plotted in panels (**A**,**B**), respectively. In panel (**C**), the 10 ng/mL M22/100 ng/mL IGF-1 treated HOFs 3D sphenoids without or with 100 nM omidenepag (OMD) were subjected to a qPCR analysis to estimate the mRNA expression of selected ECMs (*COL*; *collagen1, 4 and 6*), ECM-regulatory genes (*LOX*: lysil oxidase, and *CTGF*: Connective Tissue Growth Factor), and inflammatory cytokines (*IL1β*: interleukin-1β), which were differently regulated between GHOFs and n-HOFs upon the administration of 100 nM OMD (Table 2). All experiments were performed in duplicate using fresh preparations, each of which consisted of 16 spheroids. Data are presented as the arithmetic mean ± standard error of the mean (SEM). ** *p* < 0.01, **** *p* < 0.001 (ANOVA followed by a Tukey’s multiple comparison test).

Since it is known that the stimulation of TSH and IGF-1 receptors are essential for the etiology [13,14,15], it would be of great interest to test whether both receptor-activated 3D n-HOFs could serve as a suitable disease model for GO. To characterize this issue, we next studied the effects of several combinations of IGF-1, M22 and OMD on the physical properties of the n-HOFs. As shown in Figure 5, M22 or IGF-1 had no effect on the physical properties of the n-HOFs. Quite interestingly, in the presence of M22 and IGF-1, in addition to OMD, a significant enlargement of the 3D n-HOFs spheroids but no significant effects on their stiffness were observed, similar to the 3D GHOFs that were treated with OMD (Figure 1 and Figure 2). To examine this issue further, the OMD-induced effects on the expression of several genes of M22/IGF-1 that were activated in 3D n-HOFs spheroids were studied. Among the six different genes between n-HOFs and GHOFs, 3D spheroids (*COL1*, *COL4*, *COL6*, *LOX*, *CTGF* and *IL1β*) and OMD exerted effects on four genes (*COL4*; no change, *COL6*; no change, *CTGF*; no change, and *IL1β*; significant up-regulation) of M22; IGF-1-stimulated, 3D n-HOFs spheroids were identical with those of the 3D GHOFs spheroids (Figure 3 and Figure 4, and Table 2). Therefore, these collective results suggest that M22 and IGF-1-stimulated, 3D n-HOFs spheroids may replicate the OMD-induced effect on GHOFs spheroids. 

### 3.4. Comparison of Several mRNA Expressions of ECM, ECM Modulators, Inflammatory Cytokines, and ER Stress-Related Genes among n-HOFs, GHOFs and M22/IGF-1-Stimulated n-HOFs 3D Spheroids

**Figure 6:**
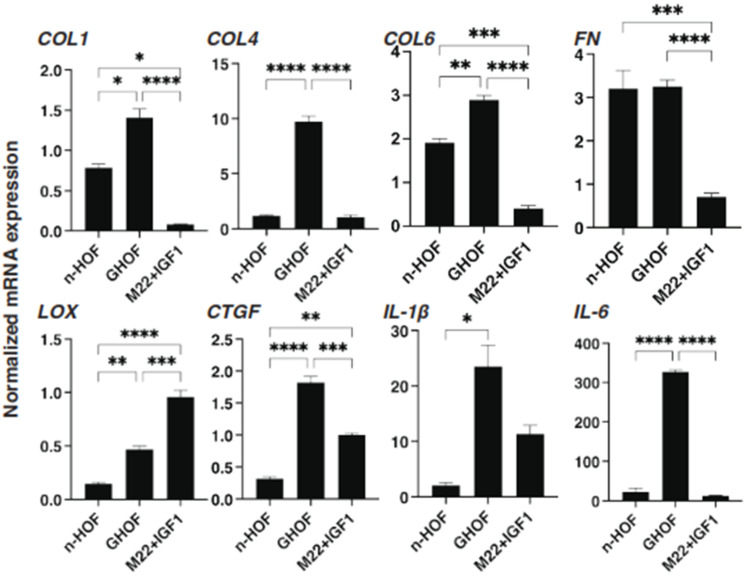
mRNA expression of selected ECMs and inflammatory cytokines among n-HOF-, GHOF- and M22/IGF-1-treated n-HOFs 3D spheroids. At Day 6, n-HOF-, GHOF- or 10 ng/mL M22/100ng/mL IGF-1-treated n-HOFs 3D spheroids were subjected to a qPCR analysis to estimate the mRNA expression of selected ECMs (*COL*; *collagen1, 4 and 6*) and inflammatory cytokines (*IL1β*: interleukin-1β). All experiments were performed in duplicate using fresh preparations, each of which consisted of 16 spheroids. Data are presented as the arithmetic mean ± standard error of the mean (SEM). * *p* < 0.05, ** *p* < 0.01, *** *p* < 0.005, **** *p* < 0.001 (ANOVA followed by a Tukey’s multiple comparison test).

**Figure 7:**
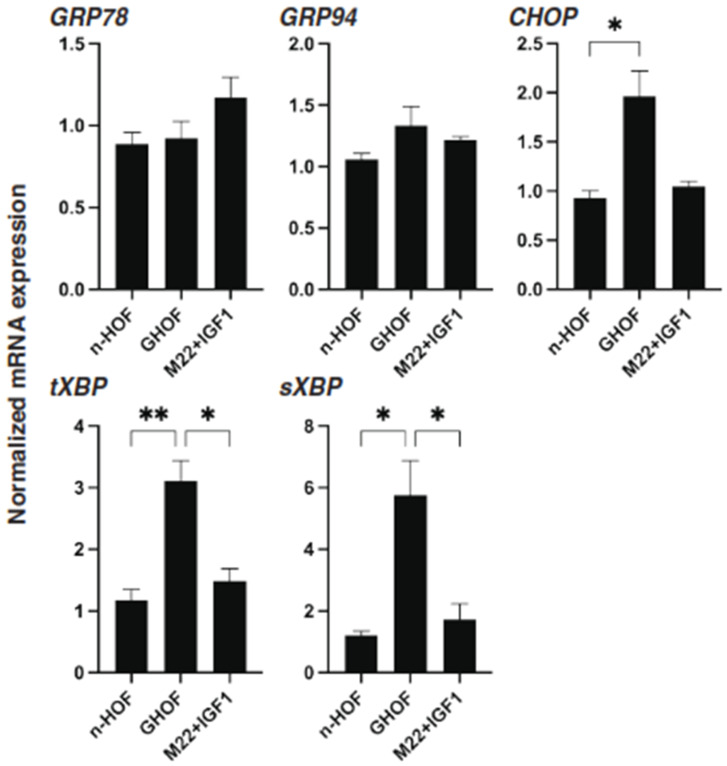
mRNA expression of ER stress-related genes among n-HOF-, GHOF- and M22/IGF-1-treated n-HOFs 3D spheroids. At Day 6, n-HOF-, GHOF- or 10 ng/mL M22/100 ng/mL IGF-1-treated n-HOFs 3D spheroids were subjected to a qPCR analysis to estimate the mRNA expression of ER stress-related genes, including major ER stress-related genes of the inositol-requiring enzyme 1 (IRE1), glucose regulator protein (GRP)78, GRP94, the X-box-binding protein-1 (XBP1), spliced XBP1 (sXBP1) and CCAAT/enhancer-binding protein homologous protein (CHOP). All experiments were performed in duplicate using fresh preparations, each of which consisted of 16 spheroids. Data are presented as the arithmetic mean ± standard error of the mean (SEM). * *p* < 0.05, ** *p* < 0.01 (ANOVA followed by a Tukey’s multiple comparison test).

To further characterize the three different 3D spheroids obtained from n-HOFs, GHOFs and M22/IGF-1-stimulated n-HOFs, the mRNA expressions of several genes, including ECM, ECM-regulatory factors, inflammatory cytokines and ER-stress related factors were compared. As shown in Figure 6 and Figure 7, most of the genes, except for FN, GRP78 and GRP94, were significantly up-regulated in the 3D GHOFs spheroids, and a significant up-regulation of LOX and CTGF, and down-regulation of COL1, COL6 and FN was observed in the M22/IGF-1-stimulated, 3D n-HOFs spheroids, as compared to the corresponding values for the 3D n-HOFs spheroids. Therefore, these collective observations indicate that the TSH and IGF-1 receptor-activated, 3D n-HOFs spheroids were physically similar to the 3D GHOFs spheroids, but their biological characteristics were quite distinct from each other. 

## 4. Discussion

The technology of 3D cell cultures recently received great attention regarding how it might structure the models of many diseases [22]. In comparison to the 2D system, this technology is potentially more advantageous for evaluating the structure of tissues close to the biological context and network of ECM proteins [23]. Since the orbital fibroblast should spread toward 3D spaces within the orbital cone during its differentiation, a 3D cell culture technology is more desirable than conventional 2D cell culture methods for this research purpose. Nevertheless, as far as we know, no related studies were conducted using a 3D cell culture technology because of their technical difficulties, although there were several studies using a 2D cell culture [24,25,26,27]. Previously, to understand the molecular pathology of GO, our group first succeeded in obtaining the 3D spheroids of n-HOF and GHOF using a 3D drop culture method, in which no scaffold was required [10]. Thereafter, using this 3D culture technique to establish a disease model of DEUS, we studied the effects by anti-glaucoma drugs on several adipocytes, including 3T3-L1 cells or n-HOFs. The results indicated that PGs and OMD induced a substantial downsizing and enlargement in the 3D spheroids by virtue of causing a significant modulation in ECM expression, in addition to the suppression of adipogenesis [12,16,17]. Therefore, since these anti-glaucoma drugs affected the physical properties of these adipose cells in addition to their adipogenesis, we rationally speculated that these drugs may have a significant effect on preadipocytes themselves. In fact, we recently reported that PGs, OMD and Rip induced different effects on GO-related human orbital fibroblasts (GHOFs) [11], and proposed that similar effects may also occur in non-GO-related human orbital fibroblasts (n-HOFs). To examine this further, we investigated this issue using a 3D drop culture of n-HOFs and found that both OMD and Rip induced a significant alteration in their physical properties and the expressions of ECM and other related genes. Interestingly, as shown in Table 2, the effects of OMD on the physical properties and the expression of some of the genes on 3D n-HOFs were quite different from 3D GHOFs [11]. In terms of the different effects between OMD and Rip, we also found that the addition of an EP2 agonist, OMD, significantly but differently modulated the Rip-induced effects on adipogenesis and the physical properties of 2D- and 3D-cultured 3T3-L1 cells [28].

In terms of the molecular pathogenesis of GO, the cross talk between the TSH receptor (TSHR), a G protein-coupled receptor (GPCR) family member, and the IGF-1 receptor (IGF-1R), tyrosine kinases, was similar to other well-established signaling mechanisms [29,30]. In fact, interactions between TSHR and IGF-1R signaling were reported to occur in primary cultures of GHOFs [14,15,31], and this signaling induced a synergistic increase in the secretion of hyaluronic acid (HA), a major component that was produced in the pathogenesis of GO [2], and for which IGF-1 induced a more than 10-fold increase in sensitivity to TSH. Based upon these observations, in studies of the pathogenesis of GO, autoantibodies that bind to and stimulate TSHR [32] and IGF-1R [33,34] were used. In the present study, to stimulate both TSHR and IGF-1R in n-HOFs, we used M22 and IGF-1 and the findings indicated that the OMD-induced effects on n-HOFs that were activated by M22/IGF-1 were almost identical to those for GHOFs, although those toward 3D n-HOFs were different from the effects induced on 3D GHOFs (Table 2). Thus, these collective findings rationally suggest that the drug reactivity by OMD may be useful in distinguishing between n-HOF and GHOF, and thus this could be applicable for screening HOFs if suitable GO models could be generated. To our knowledge, this is the first study that demonstrates the methodology of distinguishing between n-HOF and GHOF by using a 3D spheroid culture, and explores the drug-induced effect by OMD. 

Concerning the limitations of this study, (1) the gene expression profiles related to the M22/IGF-activated n-HOFs and GHOFs were poorly characterized and (2) the expression of the mRNA of several ECMs, ECM modulators, inflammatory cytokines and E stress-related factors were quite different among the n-HOFs, M22/IGF-1 stimulated n-HOFs and the GHOFs 3D spheroids. Therefore, these collective observations suggest that some factors other than the TSH and IGF-1 receptor activations may be missing when 3D GHOFs spheroids are replicated from 3D n-HOFs spheroids. At the time of writing, such additional factors have not yet been elucidated. Therefore, further studies directed toward characterizing the pharmacological and pathological aspects of OMD toward n-HOFs, M22/IGF-1-activated n-HOFs and GHOFs are required.

## Figures and Tables

**Table 1:** The sequences of the primers and Taqman probes used in the present study.

		Sequence	Exon Location	RefSeq Number
human RPLP0	Probe	5′-/56-FAM/CCCTGTCTT/ZEN/CCCTGGGCATCAC/3IABkFQ/-3′	2-3	NM_001002
	Forward	5′-TCGTCTTTAAACCCTGCGTG-3′		
	Reverse	5′-TGTCTGCTCCCACAATGAAAC-3′		
human COL1A1	Probe	5′-/56-FAM/TCGAGGGCC/ZEN/AAGACGAAGACATC/3IABkFQ/-3′	1-2	NM_000088
	Forward	5′-GACATGTTCAGCTTTGTGGAC-3′		
	Reverse	5′-TTCTGTACGCAGGTGATTGG-3′		
human COL4A1	Probe	5′-/56-FAM/TCATACAGA/ZEN/CTTGGCAGCGGCT/3IABkFQ/-3′	51-52	NM_001845
	Forward	5′-AGAGAGGAGCGAGATGTTCA-3′		
	Reverse	5′-TGAGTCAGGCTTCATTATGTTCT-3′		
human COL6A1	Forward	5′-CCTCGTGGACAAAGTCAAGT-3′	2-3	NM_001848
	Reverse	5′-GTGAGGCCTTGGATGATCTC-3′		
human FN1	Forward	5′-CGTCCTAAAGACTCCATGATCTG-3′	3-4	NM_212482
	Reverse	5′-ACCAATCTTGTAGGACTGACC-3′		
human LOX	Forward	5′-ACATTCGCTACACAGGACATC-3′	6-7	NM_002317
	Reverse	5′-TTCCCACTTCAGAACACCAG-3′		
human CTGF	Forward	5′-GAAGCTGACCTGGAAGAGAAC-3′	4-5	NM_001901
	Reverse	5′-GCTCGGTATGTCTTCATGCTG-3′		
human EPAS1	Forward	5′-AGCCTATGAATTCTACCATGCG-3′	7-8	NM_001430
	Reverse	5′-CTTTGCGAGCATCCGGTA-3′		
human IL-1β	Probe	5′-/56-FAM/AGAAGTACC/ZEN/TGAGCTCGCCAGTGA/3IABkFQ/-3′	1-3	NM_000576
	Forward	5′-CAGCCAATCTTCATTGCTCAAG-3′		
	Reverse	5′-GAACAAGTCATCCTCATTGCC-3′		
human IL-6	Probe	5′-/56-FAM/CAACCACAA/ZEN/ATGCCAGCCTGCT/3IABkFQ/-3′	4-5	NM_000600
	Forward	5′-GCAGATGAGTACAAAAGTCCTGA-3′		
human GRP78	Forward	5′-CATCACGCCGTCCTATGTCG-3′		NM_005347
	Reverse	5′-CGTCAAAGACCGTGTTCTCG-3′		
human GRP94	Forward	5′-CTGGGACTGGGAACTTATGAATG-3′		NM_003299
	Reverse	5′-TCCATATTCGTCAAACAGACCAC-3′		
human CHOP	Forward	5′-GGAGAACCAGGAAACGGAAAC-3′		NM_004083
	Reverse	5′-TCTCCTTCATGCGCTGCTTT-3′		
human tXBP	Forward	5′-AGTAGCAGCTCAGACTGCCA-3′		NM_005080
	Reverse	5′-CCTGGTTCTCAACTACAAGGC-3′		
human sXBP	Forward	5′-GGTCTGCTGAGTCCGCAGCAGG-3′		AB076384
	Reverse	5′-GGGCTTGGTATATATGTGG-3′		

**Table 2:** Comparison of physical properties, size and stiffness, and mRNA expressions of ECM and other genes between 3D GHOFs spheroid, 3D n-HOFs spheroid and M22/IGF-1-treated 3D n-HOFs spheroid in the presence of OMD or Rip.

			GHOFs*	n-HOFs	M22/IGF-1 HOFs
Physical Properties	Size	OMD	↑	(−)	↑
		Rip	↑	↑	
	Stiffness	OMD	(−)	↓	(−)
		Rip	↓	↓	
ECM genes	*COL1*	OMD	↓	(−)	(−)
		Rip	↓	(−)	
	*COL4*	OMD	(−)	↓	(−)
		Rip	(−)	(−)	
	*COL6*	OMD	(−)	↓	(−)
		Rip	(−)	(−)	
	*FN*	OMD	↓	↓	
		Rip	↓	(−)	
Other genes	*LOX*	OMD	↓	(−)	(−)
		Rip	(−)	(−)	
	*CTGF*	OMD	(−)	↓	(−)
		Rip	↓	↓	
	*IL1β*	OMD	↑	(−)	↑
		Rip	(−)	(−)	
	*IL6*	OMD	↑	↑	
		Rip	(−)	(−)	

GO; Graves’ orbitopathy, HOFs; human orbital fibroblasts, GHOFs; GO-related HOFs, n-HOFs; non-GO HOFs, OMD; omidenepag, Rip; ripasudil, *COL1*; collagen 1, *COL4*; collagen 4, *COL6*; collagen 6, *FN*; fibronectin, *LOX*; lysil oxidase, *IL1ß*; interleukin-1ß, *IL6*; interleukin-6, ↑: significant increase, ↓: significant decrease, (–): insignificant change. Data related to GHOFs* were taken from our previous study [11]. Diverse responses between 3D GHOFs spheroid and 3D n-HOFs spheroid are designated by highlight, and compared with those of M22/IGF-1-treated 3D n-HOFs spheroids.

## Data Availability

All are available in the paper.
